# Clinical Efficacy of Nutritional Supplements in Atopic Dermatitis: Systematic Review

**DOI:** 10.2196/40857

**Published:** 2023-11-29

**Authors:** Isaac Weber, Emily Woolhiser, Noah Keime, Margaret Wasvary, Madeline J Adelman, Torunn E Sivesind, Robert P Dellavalle

**Affiliations:** 1 Mercy Hospital St Louis St Louis, MO United States; 2 Kansas City University College of Osteopathic Medicine Kansas City, MO United States; 3 School of Medicine University of Colorado Aurora, CO United States; 4 Wayne State University School of Medicine Detroit, MI United States; 5 Department of Dermatology University of Colorado Aurora, CO United States; 6 Department of Epidemiology Colorado School of Public Health Denver, CO United States; 7 Dermatology Service Rocky Mountain Regional VA Medical Center Aurora, CO United States

**Keywords:** atopic dermatitis, eczema, nutrition, dietary supplement, oral supplement, vitamin, probiotic, dermatology, over the counter

## Abstract

**Background:**

Atopic dermatitis (AD), also known as eczema, is a chronic inflammatory skin condition that presents with symptoms of intense pruritus, dryness, and erythema. Dissatisfaction with first-line therapies for AD, the desire to avoid steroids, and the extreme cost of effective biologics have created a demand for alternative treatment options such as oral vitamins and nutritional supplements.

**Objective:**

The purpose of this review was to assess the effectiveness of oral nutritional supplements, pre- and probiotics, and vitamin deficiencies and supplements on AD symptomology and clinical course.

**Methods:**

We searched Scopus, PubMed, and MEDLINE (Ovid interface) for English-language articles published between 1993 and 2023. The final search was conducted on June 22, 2023. The search terms comprised the following: “(Atopic Dermatitis or Atopic Eczema) AND (supplement OR vitamin OR mineral OR micronutrients OR Fish Oil OR Omega Fatty Acid OR Probiotics OR Prebiotics OR apple cider vinegar OR collagen OR herbal OR fiber).”

**Results:**

A total of 18 studies—3 (17%) evaluating vitamins, 4 (22%) evaluating herbal medicine compounds, 2 (11%) evaluating single-ingredient nutritional supplements, and 9 (50%) evaluating pre- and probiotics—involving 881 patients were included in this review.

**Conclusions:**

Overall, there is weak evidence to support any one nutritional supplement intervention for the alleviation of AD symptoms. Multiple trials (4/18, 22%) showed promise for supplements such as Zemaphyte, kefir, and freeze-dried whey with *Cuscuta campestris* Yuncker extract. The most evidence was found on the effectiveness of probiotics on the clinical course of AD. *Lactiplantibacillus plantarum*, *Ligilactobacillus salivarius*, and *Lactobacillus acidophilus* specifically showed evidence of efficacy and safety across multiple studies (6/18, 33%). However, larger, more extensive randomized controlled trials are needed to determine the true effectiveness of these supplements on the broader population.

**Trial Registration:**

PROSPERO CRD42023470596; https://tinyurl.com/4a9477u7

## Introduction

### Background

Atopic dermatitis (AD), also known as eczema, is a chronic inflammatory skin condition that presents with symptoms of intense pruritus, dryness, and erythema. In the acute phase, inflammatory changes are dominated by edema, vesicles, and weeping skin lesions, which lead to chronic cutaneous manifestations, including thickening of the skin and fibrosis [1]. AD has 2 classifications: intrinsic (endogenous) and extrinsic (exogenous). Extrinsic AD accounts for approximately 80% of patients and is characterized by early onset and elevated levels of total serum immunoglobulin E (IgE). Sensitization to IgE is fundamental to the pathogenesis of extrinsic AD [2]. Conversely, intrinsic AD is associated with normal total serum IgE levels and the absence of IgE-mediated sensitization [2].

The pathogenesis of AD has been well studied—the acute phase is characterized by a T helper cell type 2 (Th-2) dominant response triggered by the cytokines interleukin (IL)-4, IL-5, and IL-13. This cascade results in increased IgE synthesis, mast cell activation, and eosinophil stimulation [2]. In addition, keratinocytes in the epidermis of patients with AD produce thymic stromal lymphopoietin, a cytokine that promotes the activation of dendritic cells that subsequently produce more cytokines, resulting in amplification of the Th-2 allergenic response [2].

Filaggrin, a structural protein, plays a vital role in protecting the skin barrier. Mutations or deficiencies in filaggrin can lead to the loss of transepidermal water and cause detrimental changes in the pH of the skin. These changes make the skin barrier increasingly vulnerable to environmental allergens and have been shown to be major predisposing factors for AD [3].

Given this, treatment for AD includes the restoration of the factors necessary to maintain the epidermal barrier function. Dissatisfaction with first-line therapies for AD, the desire to avoid steroids, and the extreme cost of effective biologics create a demand for alternative treatment options such as oral vitamins and nutritional supplements. Oral supplements are a growing industry garnering the attention of patients and medical professionals alike. The market for oral supplements is flooded with a wide range of products offering broad availability and convenience supported by a spectrum of customer testimonials. Currently, these supplements are regulated as food rather than drugs under the governance of the Food and Drug Administration.

### Objectives

The “food” classification allows these products to become available to customers without proof of meeting the efficacy and safety standards required of pharmaceuticals to enter the market. To ensure that physicians are providing evidence-based advice regarding adjunctive over-the-counter treatment options and that they are able to educate patients when inevitable questions arise regarding supplementation, it is paramount that they understand the utility, safety, and knowledge gaps associated with common dietary supplements. The purpose of this review was to assess the effectiveness of oral nutritional supplements, pre- and probiotics, and vitamin deficiencies and supplements on AD symptomology and clinical course.

## Methods

The PRISMA (Preferred Reporting Items for Systematic Reviews and Meta-Analyses) statement was used to conduct this study. Case-control studies, cross-sectional studies, cohort studies, and randomized controlled trials (RCTs) with ≥5 participants conducted on individuals aged >12 years were included. We excluded case reports, case series, review papers, and studies with participants aged <13 years. Eligible interventions included any study evaluating oral vitamins, minerals, or nutritional supplements in relation to AD and any vitamin, mineral, or nutritional supplement intervention for AD. Eligible methodologies to measure changes in AD severity included the Scoring Atopic Dermatitis (SCORAD) index, Eczema Area and Severity Index (EASI), Rajka-Langeland scores, Investigator Global Assessment (IGA) score, Three-Item Severity (TIS) score, Dermatology Life Quality Index (DLQI), subjective AD severity, and AD severity evaluated by a physician.

We searched Scopus, PubMed, and MEDLINE (Ovid interface) for English-language articles published between 1993 and 2023. The final search was conducted on June 22, 2023. The search terms comprised the following: “(Atopic Dermatitis or Atopic Eczema) AND (supplement OR vitamin OR mineral OR micronutrients OR Fish Oil OR Omega Fatty Acid OR Probiotics OR Prebiotics OR apple cider vinegar OR collagen OR herbal OR fiber).”

**Figure 1:**
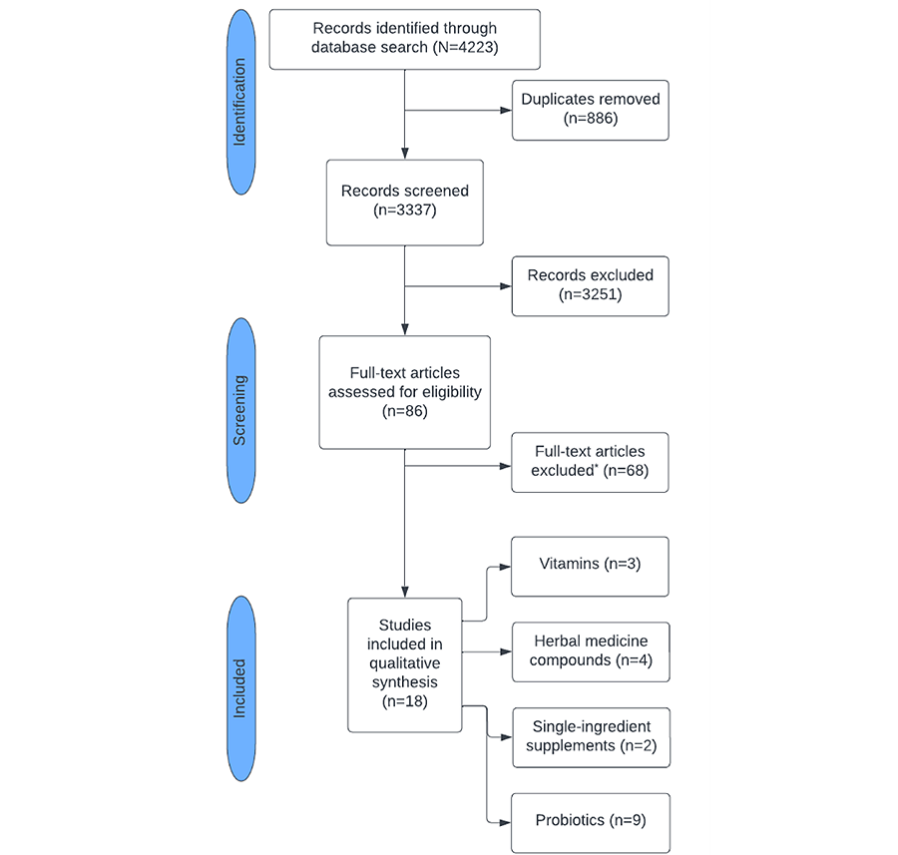
Study selection. *All full-text articles were excluded due to lack of inclusion criteria eligibility.

Literature search results were exported to CADIMA (Julius Kühn-Institut) to remove duplicates and review articles. A total of 3337 unique studies were screened and assessed for eligibility by 2 reviewers working independently. Disagreements were resolved through a third reviewer’s decision. After applying the inclusion and exclusion criteria, 18 studies (n=3, 17% evaluating vitamins; n=4, 22% evaluating herbal medicine compounds; n=2, 11% evaluating single-ingredient nutritional supplements; and n=9, 50% evaluating pre- and probiotics) involving 881 patients were selected for inclusion (Figure 1).

## Results

### Overview

Table 1 summarizes the included studies’ findings and evidence levels according to the ratings of the Oxford Centre for Evidence-Based Medicine [4]. The levels of evidence are defined as level 1 (randomized trials or systematic reviews of randomized trials, cross-sectional studies, inception cohort studies, or nested case-control studies), level 2 (a systematic review of surveys, randomized trials, individual cross-sectional studies with consistent reference standards and blinding, inception cohort studies, or [exceptional] observational studies with dramatic effect), level 3 (cohort studies, local nonrandom sample, nonconsecutive studies, or studies without a consistently applied reference standard), level 4 (case series, case-control studies, or historically controlled studies), and level 5 (mechanism-based reasoning). Level 1 represents evidence generally considered to be stronger, and level 5 represents evidence generally considered to be weaker. The Cochrane Collaboration tool for assessing the risk of bias was used to evaluate each study [5].

**Table 1:** Included studies (N=18).

Study		Study design	Participants	Sample size	Intervention	Findings	Limitations	Evidence level [4]
**Vitamins/minerals**								
	Hata et al [6]	Randomized double-blind placebo-controlled trial	Moderate to severe AD^a^ determined via a Rajka-Langeland score of 4-9; mild psoriasis determined using the Psoriasis Area and Severity Index; nonatopic participants	76	Participants were randomized to receive either 4000 IU^b^ of vitamin D3 or a placebo daily for 21 days; AD assessed using the EASI^c^ and Rajka-Langeland score	No correlation between baseline 25(OH)D^d^ levels and baseline Rajka-Langeland scores (*r*=0.04; *P*=.85); no change in mean EASI score was observed following supplementation; adverse events: none	Confounding variables and small sample size	1
	Javanbakht et al [7]	Randomized double-blind placebo-controlled trial	Patients with AD aged 13-45 years diagnosed based on the Hanifin and Rajka criteria	52	Participants were randomly divided into 4 groups receiving the following daily for 60 days: vitamin D3 and E placebos (group P), 1600 IU of vitamin D3 plus a vitamin E placebo (group D), 600 IU of all-racemic α-tocopherol plus a vitamin D3 placebo (group E), and 1600 IU of vitamin D3 plus 600 IU of all-racemic α-tocopherol (group DE); AD assessed using the SCORAD^e^ and topical steroid use (recorded in times per day)	Compared with baseline SCORAD score (*P*=.004): group D—reduction of 34.8%, group E—reduction of 35.7%, group DE—reduction of 64.3%, and group P—reduction of 28.9%; compared with baseline objective symptoms (*P*=.002): group D—improvement of 38.2%, group E—improvement of 30.1%, group DE—improvement of 64.3%, and group P—improvement of 31.04%; the change in intensity was 25.2%, 36.8%, 23%, and 62% for groups P, D, E, and DE, respectively (*P*=.001)	Confounding variables (topical steroid use per day was recorded, but the potency of the steroid was not) and small sample size	1
	Amestejani et al [8]	Randomized double-blind placebo-controlled trial	Patients with AD aged ≥14 years diagnosed at an outpatient dermatology clinic	60	Participants were randomly divided into 2 groups (1600 IU of vitamin D and a placebo) and treated once daily for 60 days; AD assessed using the SCORAD and TIS^f^ administered by the same physician before and after the trial	SCORAD score significantly improved in the vitamin D group for the following metrics: mean patients SCORAD, patients with mild, moderate, and severe AD (*P*<.05); TIS value significantly improved in the vitamin D group for the following metrics: mild, severe, and total patients TIS value (*P*<.05); no improvement in SCORAD or TIS scores in the placebo group (*P*<.05)	Small sample size, confounding variables, and study design (lack of longitudinal design)	1
**Herbal medicine compounds**								
	Latchman et al [9]	Open case-control trial	Adult patients with moderate to severe recalcitrant AD; controls: age and sex matched with no history of atopy	48	Treatment with Zemaphyte, a standardized formulation of plant materials containing *Ledebouriella seseloides*, *Potentilla chinensis*, *Clematidis* *armandii*, *Rehmannia* *glutinosa*, *Paeonia lactiflora*, *Lophatherum gracile*, *Dictamnus dasycarpus*, *Tribulus terrestris*, *Glycyrrhiza* *uralensis*, and *Schizonepeta tenuifolia* for 8 weeks; AD assessed using erythema and surface damage scores	Significant improvement in erythema and surface damage scores from baseline in the treatment group (*P*<.001)	Confounding variables and small sample size	4
	Sheehan et al [10]	Double-blind placebo-controlled crossover trial follow-up	Patients with recalcitrant AD aged 16-65 years diagnosed via recognized clinical criteria who had previously completed a placebo-controlled trial of Zemaphyte	28	Participants were divided based on their choice into either group 1 (continue with 200 mL of Zemaphyte [daily for 3 months then reduce the frequency of treatments to alternate daily if the clinical assessment of disease activity improved by 70% from the baseline assessment and to every third day if there was a >90% improvement] for 1 year) or group 2 (discontinue Zemaphyte); AD assessed using erythema and surface damage (papulation, vesiculation, scaling, excoriation, and lichenification) with a standardized scoring system	The geometric mean scores for erythema and surface damage of patients were as follows: group 1—149 (95% CI 133-177) and 151 (95% CI 133-177), respectively, for month 0; 6.83 (95% CI 2.15-21.7) and 6.09 (95% CI 2.07-18.1), respectively, for month 2; and 11 (95% CI 5.77-21.1) and 8.92 (95% CI 4.67-17.0), respectively, for month 12; group 2—163 (95% CI 147-181) and 146 (95% CI 123-172), respectively, for month 0; 5.37 (95% CI 1.35-17.3) and 5.4 (95% CI 1.47-19.9), respectively, for month 2; and 53 (95% CI 21.3-132) and 55.3 (95% CI 22.7-135), respectively, for month 12; significant difference in erythema (*P*=.006) and surface damage (*P*=.002) after 12 months between groups 1 and 2; adverse events: transient nausea and abdominal distension, mild laxative effect in more than one-third of patients in group 1, no anomalous hematological or biochemical values (full blood count, serum bilirubin, aspartate aminotransferase, alkaline phosphatase, albumin, urea and electrolytes, creatinine, calcium and phosphate, glucose, and creatinine phosphokinase)	Small sample size, confounding variables, and open nature of the study introducing bias	2
	Alves et al [11]	Controlled crossover intervention study	Adults aged 19-56 years with AD; controls: healthy adults	52	Daily consumption of 100 mL of kefir for 8 weeks; AD assessed using the SCORAD	Significant decrease in SCORAD scores in the intervention group at 8 weeks compared with the control group (*P*<.001); significant decrease in SCORAD scores in the intervention group at 8 weeks compared with week 0 (*P*<.05); adverse effects: none	Absence of a double-blind placebo-controlled design, small sample size, and uncontrolled confounders	2
	Mehrbani et al [12]	Randomized double-blind placebo-controlled clinical trial	Adults aged >18 years diagnosed with moderate to severe AD using the Hanifin and Rajka criteria	52	Participants were randomized to receive 30 g of freeze-dried whey powder with 2 g of spray-dried water extract of *Cuscuta campestris* Yuncker (field dodder) or a placebo daily for 15 days, with follow-up at 15 days after treatment ended; AD assessed using self-reported pruritus and sleep disturbances; safety profile: anorexia (54.1%) and GI^g^ upset (16.6%) and no abnormalities in liver and kidney function tests, blood cell count, blood pressure, or body weight	15 days: significant improvement in pruritus in the treated group compared with the placebo group (**P*<*.001); 15 days: no significant improvement in sleep disturbance in the treated group compared with the placebo group (*P*=.09); 30 days: significant improvement in pruritus in the treated group compared with the placebo group (*P*<.001); 30 days: significant improvement in sleep disturbance in the treated group compared with the placebo group (*P*=.005)	Short study period, small sample size, and confounding factors	1
**Single-ingredient supplements**								
	Kawamura et al [13]	Double-blind placebo-controlled clinical trial	Adults with mild to moderate AD diagnosed according to the criteria of the Japanese Dermatological Association	112	200 mg of GLA^h^ (18:3n-6)-enriched oil extracted from the *Mucor circinelloides* fungus for 4 weeks with 4 weeks of follow-up; AD evaluated using VAS^i^, range and frequency of pruritus, and observations of skin manifestations graded by a physician (erythema, papules, crusting, nodules, lichenification, area of eruption, or the sum of these items)	Compared with baseline: VAS score significantly improved at week 8 in the intervention group (*P*<.05); compared with baseline: pruritus intensity and frequency of nocturnal itching significantly improved at weeks 4 and 8 in the intervention group (*P*<.05); no significant changes in VAS score, pruritus intensity, or frequency of nocturnal itching were observed in the control group; no significant differences were found between the groups in the judgment of skin manifestations as graded by a physician; no significant differences in topical treatment were observed in either group during the 8 weeks; adverse events: none	Confounding factors and inclusion of only mild to moderate AD	1
	Callaway et al [14]	Randomized controlled single-blind crossover study	Patients aged 25-60 years with AD and a BMI of <30	20	30 mL of cold-pressed hempseed oil daily for 4 weeks followed by a 4-week wash-out period and 4 weeks of olive oil (or vice versa); AD evaluated using patient ratings of atopic symptoms and medication use	Compared with baseline: subjective decreases in skin dryness (*P*=.03) and pruritus (*P*=.02) were statistically significant in the intervention group; compared with baseline: use of medication for AD significantly decreased in the intervention group (*P*=.02); no significant improvement in any metric was observed in the control group; no significant difference was found between the intervention and control groups in any metric; adverse events: none	Small sample size and short study period	1
**Probiotic**								
	Moroi et al [15]	Prospective randomized double-blind placebo-controlled parallel-group comparative study	Adults aged 20-65 years with mild to moderate AD diagnosed according to the criteria of the Japanese Dermatological Association	34	100 mg (2 × 10^11^ bacteria) of heat-killed *Lacticaseibacillus* *paracasei* K71 daily for 12 weeks; AD assessed using skin severity scores, VAS and QOL^j^ impairment scores (Skindex-16 questionnaire), and topical medication use	Skin severity scores (no significant difference between the groups) decreased significantly from baseline by 18.6% (*P*<.05) at week 8 and 27.1% (*P*<.01) at week 12 in the intervention group, and there was no significant decrease in the placebo group; VAS score (no significant difference between the groups) decreased significantly from baseline by 23% (*P*=.03) at week 4 with no significant improvement at weeks 8 (*P*=.06) or 12 (*P*=.35) in the intervention group, and there was no significant decrease in the placebo group; QOL impairment scores (no significant difference between the groups) decreased significantly from baseline by 28% (*P*<.05) at week 4, by 36.1% at week 8 (*P*<.01), and by 29.3% at week 12 (*P*<.05) in the intervention group and decreased significantly from baseline by 28.3% (*P*<.01) at week 4, by 42.5% at week 8 (*P*<.01), and by 41.9% at week 12 (*P*<.01) in the placebo group; no significant difference between the intervention and control groups in topical medication use; adverse events related to the intervention: none	Small sample size and confounding factors	1
	Prakoeswa et al [16]	Randomized double-blind placebo-controlled trial	Adults aged >14 years with mild to moderate AD according to the Hanifin and Rajka criteria and serum IgE^k^ levels of >100 IU/mL	30	2240 g (20^10^ CFU^l^) daily of a probiotic microencapsulation of *Lactiplantibacillus plantarum* IS-10506 for 8 weeks; AD assessed using the SCORAD	SCORAD significantly improved in the intervention group compared with the control group after 8 weeks (*P*=.002)	Small sample size, confounding factors, and short observation period	1
	Fang et al [17]	Placebo-controlled trial	Adult patients with AD evaluated by a dermatologist	109	Patients were randomly divided into 4 groups receiving a placebo, 10^9^ CFU of *Bifidobacterium bifidum* F35 CCFM16, oligosaccharide, or 10^9^ of *L plantarum* CCFM8610 for 8 weeks; AD assessed using the SCORAD and DLQI^m^	After 8 weeks of intervention, patients in the CCFM8610 group had a significantly improved SCORAD score compared with their baseline values (*P*<.05); no significant improvement was noted in the placebo, oligosaccharide, and CCFM16 groups; adverse events: none	Confounding factors	2
	Drago et al [18]	Randomized double-blind placebo-controlled trial	Adults aged 18-46 years with moderate to severe AD	38	Twice-daily 1 × 10^9^ CFU/g of *Ligilactobacillus salivarius* LS01-DSM 22775 for 16 weeks; AD assessed using the SCORAD and DLQI	SCORAD: significant reduction in the probiotic-treated group (T0: 27.57, SD 3.4 vs T16: 13.14, SD 0.27; *P*<.001) and no significant improvement in the placebo group; DLQI: significant improvement after 8 weeks of intervention (T8), which was maintained 4 weeks after the end of treatment (T20; T0: 8.28, SD 1.79 vs T8: 4.57, SD 1.11 and *P*=.02; T0: 8.28, SD 1.79 vs T16: 4.42, SD 0.27 and *P*=.04; T0: 8.28, SD 1.79 vs T20: 3.71, SD 0.27 and *P*=.02); no significant improvement in the placebo group; adverse events: none	Small sample size and confounding factors	1
	Drago et al [19]	Prospective controlled pilot trial	Adult patients with AD aged 25-63 years with predominant rough and fissured skin as well as pruritus for at least 2 months and diagnosed using the Hanifin and Rajka criteria	25	Once-daily freeze-dried mixture of 5 × 10^9^ CFU per sachet of *L salivarius* LS01, *Streptococcus thermophilus* ST10 at 2 × 10^9^ CFU per sachet, and tara gum (125 mg) for 4 weeks; AD assessed using the SCORAD	SCORAD score significantly improved in the active group from baseline after 4 weeks (*P*<.001); no significant improvement in SCORAD score in the placebo group after 4 weeks; after 1 month of treatment, the SCORAD index in the intervention group was significantly lower than in the placebo group (*P*=.02); adverse events: none	Small sample size, lack of follow-up data after probiotic discontinuation, and confounding factors	2
	Litus et al [20]	Open controlled randomized trial	Adults with AD diagnosed using the European Academy of Dermatology and Venereology recommendations	37	Treatment with fluticasone propionate 0.005% ointment twice daily, emollient twice daily, and a probiotic capsule containing *Lactobacillus acidophilus* and *Bifidobacterium animalis lactis* 2 times daily; participants in this study were divided into groups based on total IgE levels, with the exogenous or IgE-dependent AD group comprising participants with a total IgE level of >100 IU/mL and the endogenous or IgE-independent AD group comprising participants with a total IgE level of <100 IU/mL; patients were then further stratified according to genotypes of the CD14 receptor gene, CC and TT; each subset of participants received either fluticasone propionate 0.005% ointment twice daily and emollient twice daily or fluticasone propionate 0.005% ointment twice daily, emollient twice daily, and a probiotic capsule containing *L acidophilus* and *B animalis lactis* 2 times daily; AD assessed using the SCORAD and DLQI	SCORAD in the exogenous AD group: intervention group had a significant improvement from baseline at week 4 (*P*=.001) for the CC genotype and a significant improvement from baseline at week 4 (*P*=.02) for the TT genotype, and there were no significant differences in the control group; SCORAD in the endogenous AD group: intervention group had a significant improvement from baseline at 4 weeks (*P*=.006) for the CC genotype, the control group had a significant improvement from baseline at 4 weeks (*P*=.04) for the CC genotype, and the intervention group had a significant improvement from baseline at 4 weeks (*P*=.01) for the TT genotype; SCORAD score was significantly lower (*P*=.02) in patients with the CC genotype who received standard treatment with probiotics compared with other groups; no other SCORAD intergroup comparisons were significant; DLQI in the exogenous AD group: intervention group had a significant improvement from baseline at week 4 (*P*=.001) for the CC genotype, the control group had a significant improvement from baseline at 4 weeks (*P*=.04) for the CC genotype, and the intervention group had a significant improvement from baseline at week 4 (*P*=.02) for the TT genotype; DLQI in the endogenous AD group: intervention group had a significant improvement from baseline at 4 weeks (*P*=.03) for the CC genotype, the control group had a significant improvement from baseline at 4 weeks (*P*=.03) for the CC genotype, and the intervention group had a significant improvement from baseline at 4 weeks (*P*=.03) for the TT genotype; DLQI was significantly lower (*P*=.01) in patients with the CC genotype who received standard treatment with probiotics compared with other groups; no other DLQI intergroup comparisons were significant	Small sample size and confounding factors	1
	Yamamoto et al [21]	Placebo-controlled double-blind parallel-group comparison study	Patients aged >16 years with mild to moderate AD defined according to the Japanese Dermatological Association criteria	57	20.7 mg once daily of heat-killed *L acidophilus* L-92 for 24 weeks; AD assessed using the IGA^n^, EASI, and SCORAD	IGA: significant improvement at 16 (*P*=.03), 20 (*P*=.03), and 24 weeks (*P*<.001) when compared with the placebo; EASI: significant improvement at 8 (*P*=.05) and 24 weeks (*P*=.01) when compared with the placebo; SCORAD: significant improvement at 8 (*P*=.02), 12 (*P*=.01), 16 (*P*=.02), 20 (*P*=.01), and 24 weeks (*P*<.001) when compared with the placebo; adverse events: none	Small sample size and confounding factors	1
	Wang et al [22]	Cohort pilot study	Adults aged 18-73 years with mild to severe chronic AD (>3 years)	41	Once-daily probiotic mixture of 2 × 10^10^ CFU per capsule of *Lacticaseibacillus rhamnosus* GG, *L acidophilus* GKA7, *Lactococcus lactis* GKL2, *Lacticaseibacillus casei* GKC1, *L paracasei* GKS6, *B bifidum* GKB2, and *B animalis lactis* GKK2; 10 mg per capsule of postbiotic heat-killed *L* *plantarum*; and 22 mg per capsule of triple prebiotics containing inulin for 2 months; AD assessed using the EASI	EASI was significantly reduced (*P*<.001) after 8 weeks of intervention regardless of baseline disease severity, although the minimal clinically important difference was not reached; more patients with mild AD significantly improved (82.4%) after the intervention compared with patients with severe AD (41.7%; *P*<.001)	Lack of a control or a placebo group, small sample size, and confounding factors (inclusion of numerous probiotic strains)	3
	Matsumoto et al [23]	Double-blind placebo-controlled crossover study	Adult patients aged >15 years with moderate AD diagnosed by a clinician	10	100 g of yogurt fermented with 5.2 × 10^7^ CFU/g of *B animalis* *lactis* LKM512 and 4.7 × 10^8^ CFU/g of *Lactobacillus delbrueckii* *bulgaricus* LKM175 and *S thermophilus* LKM1742 daily; participants received yogurt or placebo for 4 weeks followed by a 4-week wash-out period and 4 weeks of yogurt or placebo; AD assessed using a questionnaire evaluating subjective symptoms	Intervention: 40% of participants experienced improvement in pruritus, and 37.5% of participants experienced improvement in burning; placebo: 10% of participants experienced improvement in pruritus, and 25% of participants experienced improvement in burning	Small sample size and lack of clarity as to whether the results were significant	1

^a^AD: atopic dermatitis.

^b^IU: international unit.

^c^EASI: Eczema Area and Severity Index.

^d^25(OH)D: 25-hydroxyvitamin D.

^e^SCORAD: Scoring Atopic Dermatitis.

^f^TIS: Three-Item Severity.

^g^GI: gastrointestinal.

^h^GLA: gamma-linolenic acid.

^i^VAS: visual analog scale.

^j^QOL: quality of life.

^k^IgE: immunoglobulin E.

^l^CFU: colony-forming unit.

^n^IGA: Investigator Global Assessment.

### Vitamins

A total of 17% (3/18) of the studies evaluated either the relationship between levels of serum 25-hydroxyvitamin D (25(OH)D) and AD severity or vitamin D3 and vitamin E as an intervention for AD. Hata et al [6] (N=76) conducted a randomized, placebo-controlled, double-blind trial that examined whether 25(OH)D levels correlated with AD severity; in addition, this study evaluated the effect of 4000 international units (IU) of oral vitamin D3 for 21 days on patients with moderate to severe AD. At the onset of the trial, 25(OH)D levels were found to be similar between patients with AD and control patients, and no correlation was found between baseline 25(OH)D levels and baseline AD severity evaluated using Rajka-Langeland scores [6]. Participants were randomized to receive either a placebo or 4000 IU of vitamin D3, and no difference was found in the mean EASI score between the groups after supplementation [6].

Javanbakht et al [7] (N=52) conducted a separate randomized, placebo-controlled, double-blind trial evaluating the effect of 1600 IU of vitamin D3 for 60 days as an intervention for AD. This study found a significant reduction in baseline SCORAD scores after 60 days in the vitamin D3 group (34.8%; *P*=.004); however, the difference between the placebo and vitamin D3 groups was not significant [7]. The change in objective symptoms and intensity in the intervention group was found to be 38.2% and 36.8%, respectively, compared with a more modest improvement of only 31.04% and 25.2% in the placebo group (*P*=.001 and *P*=.002, respectively) [7]. No association was found between serum 25(OH)D levels and SCORAD score [7].

Another randomized, double-blind, placebo-controlled trial by Amestejani et al [8] (N=60) also evaluated the effects of 1600 IU of oral vitamin D3 daily for 60 days on patients with AD. This study found significant improvements in SCORAD and TIS values in patients with mild and severe AD after 60 days of the intervention. In addition, an improvement in mean SCORAD and total TIS values was found in the intervention group after 60 days compared with baseline (*P*<.05) [8]. No significant improvement in either metric was found in the placebo group [8].

Javanbakht et al [7] (N=52) conducted a randomized, double-blind, placebo-controlled trial evaluating the effect of 600 IU of all-racemic α-tocopherol (Vitamin E) daily for 60 days on AD severity. Patients receiving vitamin E showed a significant reduction in SCORAD scores compared with baseline (35.7%; *P*<.001) and a more drastic reduction compared with the placebo group (28.9%). However, objective symptoms and intensity did not significantly improve when compared with the placebo group [7].

This study also evaluated the effects of a combined regimen of 600 IU of vitamin E and 1600 IU of vitamin D3 daily for 60 days [7]. This group showed a significant and marked improvement in SCORAD scores (64.3%; *P*<.005) compared with baseline, with the vitamin D and E groups showing reductions in severity to lesser degrees (34.8% and 35.7%, respectively) [7]. This study did find that topical steroid use as a class decreased in the vitamin D group (66.8%), the vitamin E group (70.2%), and the combined vitamin D and E group (88.7%), with use decreasing in the placebo group by only 37.5% (*P*=.05) [7].

Overall, there is minimal evidence supporting the efficacy of vitamin D or E for the treatment of AD. Any significant difference elicited in these studies was only found in relation to baseline severity, with no study showing a significant intergroup difference between the intervention and placebo groups. Larger trials with higher power are required to determine the true efficacy of combined vitamin D and E supplementation.

### Herbal Medicine Compounds

A total of 22% (4/18) of the studies evaluated the effects of herbal medicine compounds on AD symptoms. Mehrbani et al [12] (N=52) conducted a randomized, double-blind, placebo-controlled clinical trial evaluating the efficacy of 30 g of freeze-dried whey powder with 2 g of freeze-dried *Cuscuta campestris* Yuncker (field dodder) extract daily on patients with moderate to severe AD for 15 days. This study found significant improvements in subjective symptoms, specifically pruritus and sleep disturbance, in the treatment group when compared with the control group at 30 days (*P*<.001 in both cases) [12].

The side effects noted by participants in the treatment group included anorexia (54.1%) and mild gastrointestinal discomfort (16.6%) [12]. No other side effects were reported, and no dropouts resulted from these symptoms [12].

A placebo-controlled crossover trial by Alves et al [11] (N=52) evaluated the effect of daily consumption of 100 mL of kefir for 8 weeks in patients with AD. Importantly, these researchers conducted a survey evaluating the eating habits of the study participants and found no significant difference in macronutrients or dietary habits between participants who drank the kefir and their controls, indicating similar baseline dietary characteristics between the control and treatment groups [11]. This study found that, after 8 weeks, the treatment group had a significant decrease in SCORAD scores compared with the control group (*P*<.001) [11]. In addition, paired individual comparisons exhibited significantly lower SCORAD indexes compared with baseline (*P*<.001) [11].

A double-blind, placebo-controlled crossover trial follow-up (the initial study did not fit within our date parameters) by Sheehan et al [10,24] (N=28) evaluated the efficacy of a standardized formulation of plant materials known as Zemaphyte (*Ledebouriella seseloides, Potentilla chinensis, Clematidis armandii, Rehmannia glutinosa, Paeonia lactiflora, Lophatherum gracile, Dictamnus dasycarpus, Tribulus terrestris, Glycyrrhiza glabra*, and *Schizonepeta tenuifolia*) on patients with recalcitrant AD. Participants were administered 200 mL of Zemaphyte solution once daily for 3 months and then once daily, once every other day, or once every third day depending on disease severity for the next 9 months [24]. This study found significant improvement in erythema and surface damage (*P*=.006 and *P*=.002, respectively) at 12 months in the treatment group when compared with the control group [24]. Of note, the original 2-month study found no significant difference in AD symptom improvement between the treatment and control groups [10]. No abnormalities in biochemical profiles were elicited throughout the 12-month period, but side effects noted by participants in the treatment group included transient nausea and abdominal distension [24].

An open case-control study by Latchman et al [9] (N=48) also evaluated the effects of 8 weeks of Zemaphyte on patients with moderate to severe recalcitrant AD. This study found significant improvement in erythema and surface damage in patients after 8 weeks of the intervention (*P*<.001) [9].

Overall, these studies support the short-term safety and efficacy of supplements such as Zemaphyte and kefir for alleviating subjective AD symptoms, with significant intergroup differences elicited in validated AD metrics. Larger studies are needed to confirm these findings and the impressive results obtained by these small, controlled trials.

### Single-Ingredient Supplements

A total of 11% (2/18) of the trials assessed single-ingredient nutritional supplements as an intervention for AD. A double-blind controlled trial conducted by Kawamura et al [13] (N=120) studied the effects of 200 mg of gamma-linolenic acid (GLA; 18:3n-6) supplementation on patients with mild to moderate AD daily for 4 weeks. This study found significant improvement in pruritus and visual analog scale (VAS) scores after 8 weeks compared with baseline in the treatment group; however, no significant differences were found between the treatment and control groups regarding VAS, subjective pruritus intensity, or frequency of itching after 4 weeks [13]. There was also no difference noted in physician-evaluated skin manifestations (erythema, papules, crusting, nodules, lichenification, area of eruption, or the sum of these items) [13]. No adverse effects were experienced by either group [13]. The use of steroids before and after treatment was recorded, and no changes were found in the frequency of use between the intervention and control groups [13].

Callaway et al [14] (N=20) conducted a randomized controlled, single-blind crossover trial evaluating the effects of 30 mL of hempseed oil compared with 30 mL of olive oil for 20 weeks on patients with AD [14]. This study found a decrease in the use of topical medications and an improvement in skin dryness and pruritus in the intervention group compared with baseline measurements (*P*=.02, *P*=.03, and *P*=.02, respectively); however, the difference between the intervention and control groups was not significant [14]. No significant side effects were experienced by any study participant [14].

These studies do little to provide evidence of the efficacy of GLA or hempseed supplementation on AD because of the lack of significant intergroup improvement. There is minimal evidence supporting their use for alleviating AD symptoms in adults.

### Probiotics

In total, 50% (9/18) of the studies that met the inclusion criteria evaluated the effects of probiotics on AD symptoms and clinical course. Moroi et al [15] (N=34) conducted a prospective, double-blind RCT investigating the effect of a daily dose of 100 mg (2 × 10^11^ colony-forming unit [CFU]/g) of heat-killed *Lacticaseibacillus paracasei* K71 daily for 12 weeks. Subjective skin severity scores significantly decreased from baseline in the intervention group at 8 and 12 weeks (*P*<.05 and *P*<.01, respectively), with no significant improvement noted in the placebo group [15]. However, there was no significant difference found between the intervention and placebo groups at the end of the 12 weeks for changes in skin severity score, itch score, or quality of life improvement [15]. This study also found no significant difference in the use of topical medications between the intervention and control groups over the 12-week period [15]. No severe adverse events related to the study diet were experienced [15].

Prakoeswa et al [16] (N=30) conducted a randomized double-blind controlled trial comparing 2240 g (2 × 10^10^ CFU/g) of *Lactiplantibacillus plantarum* IS-10506 with a placebo daily for 8 weeks. This study found that, after 8 weeks, the intervention group had a significantly lower SCORAD index compared with the control group (*P*=.002) [16].

Fang et al [17] (N=109) also conducted a placebo-controlled trial exploring the efficacy of 1 × 10^9^ CFU of *L plantarum* CCFM8610 or *Bifidobacterium* *bifidum* F35 CCFM16 lyophilized powder daily for 8 weeks on patients with AD. This study found that, after 8 weeks, patients taking *L plantarum* had significantly improved SCORAD scores when compared with baseline (*P*<.05) [17]. No improvement in SCORAD scores was found in any other group [17]. No significant difference was found between the groups, and no significant improvement was found in DLQI scores for any group [17]. No adverse events were experienced by any patient in any group.

Another randomized, double-blind, placebo-controlled trial by Drago et al [18] (N=38) looked at the effects of 1 × 10^9^ CFU/g of *Ligilactobacillus salivarius* LS01 on adults with moderate to severe AD daily for 16 weeks. At the end of the treatment period, this study found a significant reduction in SCORAD scores in the probiotic group only (*P*<.001) [18]. It also found significant improvement in the DLQI scores after 8 and 16 weeks of treatment, which persisted for at least 4 weeks after the cessation of treatment (*P*=.002, *P*=.004, and *P*=.002, respectively) [18]. No significant improvement in either metric at any period was found in the placebo group, and no significant adverse events were reported by either group during the 16 weeks [18].

Drago et al [19] (N=25) conducted an additional prospective, controlled pilot trial evaluating the efficacy of a freeze-dried mixture of 5 × 10^9^ CFU per sachet of *L salivarius* LS0, *Streptococcus thermophilus* ST10 at 2 × 10^9^ CFU per sachet, and 125 mg of tara gum on patients with AD for 1 month. At the end of 30 days, patients in the intervention group showed significantly improved SCORAD scores when compared with baseline (*P*<.001) [19]. In addition, at the end of the month, the SCORAD index in the intervention group was significantly lower than in the placebo group (*P*=.02) [19]. No significant adverse events were experienced by any of the participants during this study [19].

Litus et al [20] (N=37) conducted an open, controlled, randomized parallel trial evaluating the efficacy of adding a twice-daily probiotic (*Lactobacillus acidophilus and Bifidobacterium animalis* *lactis)* to standard treatment for AD (fluticasone propionate 0.005% ointment and emollients) for 4 weeks*.* Participants in this study were divided into groups based on total IgE levels, with exogenous or IgE-dependent AD classified as patients with a total IgE level of >100 IU/mL and endogenous or IgE-independent AD classified as patients with a total IgE level of <100 IU/mL [20]. Patients were further stratified according to genotypes of the CD14 receptor gene (CC and TT) [20].

This study found a significant improvement in SCORAD scores after 28 days in the exogenous AD group for both the CC and TT genotypes in patients who received probiotics in addition to topical therapy (*P*=.001 and *P*=.02, respectively) [20]. No significant difference was found in the topical treatment–only group. In patients with endogenous AD, the study found a significant improvement in SCORAD scores in all groups (those treated with additional probiotics and those not); however, the improvement in SCORAD scores was significantly higher at 28 days in both participants with endogenous and exogenous AD who received probiotics and topical therapy than in the group that received topical therapy alone (*P*=.02 and *P*=.02, respectively) [20].

This study also evaluated the change in DLQI scores and found a significant improvement in both patients with exogenous AD with the CC and TT genotypes who took probiotics in addition to topical treatment for 4 weeks and those who only used topical therapy; however, the study did find a significant difference between these 2 groups (*P*=.01) [20]. It also found a significant improvement in DLQI scores in all groups with endogenous AD (both genotypes and interventions), but no significant difference was found among any of these groups [20].

Another placebo-controlled, double-blinded, parallel-group comparison study by Yamamoto et al [21] (N=57) evaluated the effects of 20.7 mg of heat-killed and dried *L acidophilus* L-92 on AD daily for 24 weeks. No adverse effects were experienced by either the placebo or the intervention group [21]. The IGA, EASI, and SCORAD scores of the intervention group were significantly lower at weeks 8 and 24 than those of the placebo group [21]. More specifically, significant differences between the intervention and placebo groups were found at 8, 16, and 24 weeks for SCORAD scores (*P*=.02, *P*=.01, and *P*<.001, respectively); at 8 and 16 weeks for EASI scores (*P*=.05 and *P*=.09, respectively); and at 16 and 24 weeks for IGA scores (*P*=.03 and *P*<.001, respectively) [21]. The SCORAD was the first measure to improve, suggesting that subjective symptoms related to itching and lack of sleep decreased first with probiotic use.

Wang et al [22] (N=41) conducted a cohort pilot study to evaluate the effect of a probiotic mixture of 2 × 10^10^ CFU per capsule of *Lacticaseibacillus rhamnosus* GG, *L acidophilus* GKA7, *Lactococcus lactis* GKL2, *Lacticaseibacillus casei* GKC1, *L paracasei* GKS6, *B bifidum* GKB2, and *B animalis lactis* GKK2; 10 mg per capsule of postbiotic heat-killed *L plantarum*; and 22 mg per capsule of triple prebiotics with inulin on mild to severe AD for 2 months. This study found a significant improvement in the EASI scores of patients with AD after 8 weeks when compared with baseline, which did not meet the minimal clinically important difference (*P*<.001) [22]. Wang et al [22] did find that more patients with mild AD improved compared with those with severe AD (*P*<.001), possibly because of the relatively easier restoration of dysbiosis in patients with mild AD when compared with the more severely imbalanced gut flora in those with severe AD.

An additional double-blind, placebo-controlled crossover study by Matsumoto et al [23] (N=10) investigated the effect of 100 g of probiotic yogurt containing *B animalis* *lactis* LKM512 (5.2 × 10^7^ CFU/g), *Lactobacillus delbrueckii* *bulgaricus* LKM1759 (4.7 × 10^8^ CFU/g), and *S thermophilus* LKM1742 (4.7 × 10^8^ CFU/g). This study found improvement in “itch” and “burning” in 40% and 37.5% of patients in the intervention group compared with 10% and 25% in the placebo group, respectively [23].

Overall, there is weak evidence supporting the use of certain strains of probiotics for improving AD symptoms with a minimal side effect profile. *Lactobacillus acidophilus*, *L salivarius*, and *L plantarum* all significantly improved AD symptoms using validated metrics when compared with a placebo. More research is needed to determine adequate dosing, time course, and effective additives such as inulin to maximize these supplements’ benefits.

## Discussion

### Vitamins

Vitamin D is a fat-soluble vitamin obtained from diet or sun exposure and plays a crucial role in the development of bones, the regulation of calcium, and the immune response against infections [25]. Subclinical vitamin D deficiency is common, affecting >1 billion people worldwide [25]. Vitamin D plays a role in the production of cathelicidin, an antimicrobial peptide that modulates the innate immune system [26]. Cathelicidins assist in protecting the skin against infections, which are a common cause of resistance to topical steroid therapy in AD [7]. The vitamin D receptor is also present in many cell types, including keratinocytes, natural killer cells, and dendritic cells [27]. In addition, UV phototherapy is an effective treatment for severe AD, with evidence supporting phototherapy playing a role in immune suppression and the production of vitamin D [7].

A total of 11% (2/18) of the studies evaluated the association between baseline vitamin D levels and AD severity, though neither study found a correlation between the 2 [6,7]. Of the 18 studies, 3 (17%) RCTs evaluated the efficacy of daily vitamin D on AD symptoms, with the shorter trial finding no change in mean EASI scores with vitamin D supplementation [6]. The other 67% (2/3) of the trials extended the treatment period to 60 days, and both found significant improvements in SCORAD and TIS scores compared with baseline; however, these improvements were not significantly different from those in the placebo group, weakening the evidence for vitamin D as an effective intervention for AD.

The limitations of these studies include the unmeasured differing use and potency of topical steroids and AD therapy between patients. In addition, the small sample sizes make it difficult to derive adequate power to show a significant difference between the treatment and placebo groups. Longitudinal studies with variable doses of vitamin D are necessary to provide evidence of its efficacy and significant intergroup differences.

Vitamin E is a fat-soluble vitamin and an essential nutrient that acts as the primary physiological barrier antioxidant in human skin, with some studies finding an association between dietary antioxidants and atopic disease [28,29]. Higher concentrations of vitamin E intake have also been found to be associated with decreased serum IgE levels and allergen sensitization [30].

Of the 18 studies, only 1 (6%) RCT evaluated vitamin E supplementation in addition to a combined regimen of vitamin D and E (1600 IU and 600 IU, respectively) [7]. Similar to the vitamin D trials, this study found significant improvements in baseline SCORAD scores compared with the placebo group; however, these improvements were not significantly different [7]. Of note, the combined vitamin D and E group did have markedly improved SCORAD scores and objective symptoms at the end of the 60-day trial period when compared with the other intervention and placebo groups, suggesting that dual supplementation could play a role in ameliorating AD severity to a greater degree [7]. This study also found a significant decrease in topical steroid use across the participant groups, with the greatest decrease in use again occurring in the combined vitamin D and E group [7]. The singular nature of this study provides a basis for more research on vitamin supplementation for the adjunctive treatment of AD and suggests that combined supplementation could have a beneficial effect.

### Herbal Supplements

Whey is a protein derived from milk and has been suggested to possess antioxidant properties owing to its intracellular conversion of cysteine into glutathione, an intracellular antioxidant [31]. *Cuscuta campestris* Yuncker (field dodder) is a parasitic plant commonly used in traditional medicine for the treatment of epilepsy, psychosis, paralysis, and skin diseases [12]. The *Cuscuta* seed is rich in flavonoids, specifically quercetin, kaempferol, and rutin, which are therapeutic compounds shown to have immunomodulatory and anti-inflammatory effects [32]. Quercetin, in particular, reduces inflammation by inhibiting Th cytokine production and inhibiting mast cell secretion [33,34].

Of the 18 studies, 1 (6%) RCT evaluated the effects of freeze-dried whey powder and extract of *C campestris* Yuncker on AD for 15 days, finding significant improvement in pruritus and sleep disturbances in the intervention group when compared with the control group [12]. This significant improvement also persisted for 15 days after treatment was stopped [12]. The limitations of this study include the small sample size, the presence of numerous confounding factors, and the subjective nature of symptom reporting in contrast to the use of a validated scale. However, these promising results suggest the need for additional longitudinal and larger trials using validated metrics for measuring AD severity to truly determine the efficacy and safety of this supplemental therapy.

Kefir is a fermented food reported to have beneficial effects on the intestinal microbiota and improve the health of the digestive system owing to its probiotic properties [35]. There is evidence suggesting that intestinal dysbiosis can contribute to epithelial permeability because of the release of proinflammatory cytokines and immune dysregulation [36,37]. Kefir consists of a mixture of lactic acid bacteria and yeast that produce numerous bioactive compounds shown to have various beneficial effects, including anti-inflammatory and antimicrobial activity [38-40].

A single crossover study looked at the effects of daily kefir consumption on patients with AD, finding a significant improvement in SCORAD scores compared with both baseline and the control group [11]. These findings are weakened by the lack of a double-blind, placebo-controlled design, with the crossover nature of the study introducing limitations because of the long-term changes in gut microbiota that can occur and persist for longer than the study wash-out period [11]. However, the positive results of this small trial and the lack of side effects over 8 weeks provide an impetus for larger RCTs to evaluate the effectiveness of this intervention.

Of the 18 studies, 2 (11%) separate studies that met the inclusion criteria evaluated the efficacy of Zemaphyte, a standardized formulation of plant materials consisting of *L seseloides, P chinensis, C armandii, R glutinosa, P lactiflora, L gracile, D dasycarpus, T terrestris, G glabra*, and *S tenuifolia* [9,24]. This compound provokes immunologic changes and has been shown to decrease both serum-complexed IgE and serum IL-2 receptors [9]. Decreased complexed IgE prevents the binding of IgE to mast cells, B cells, eosinophils, and monocytes, reducing inflammatory molecules that can exacerbate skin damage [9]. IL-2 receptors are expressed by activated T cells, and their quantity in serum reflects surface expression; thus, this parameter is useful for monitoring T cell activation [9]. Serum-soluble IL-2 receptor levels have been shown to correlate with AD disease activity and improve with treatment using topical steroids [41].

Both studies found significant improvements in erythema and surface damage in the treatment group when compared with baseline [9,24]. The trial with a longer duration (1 year) also found a significant difference in both of these metrics in the intervention group when compared with the placebo group [24]. The results of these studies provide promising evidence regarding the efficacy of Zemaphyte in the treatment of adult AD, with larger RCTs needed to truly assess the effectiveness and safety of this supplement. The limitations of both studies include their open nature, which introduces bias as participants drop out; the small sample sizes; and the presence of a multitude of confounding factors.

### Single-Ingredient Supplements

GLA has been shown to be beneficial in improving transepidermal water loss and reversing epidermal hyperproliferation [42,43]. GLA is metabolized from linolenic acid (LA), and both GLA and LA are polyunsaturated essential omega-6 fatty acids with anti-inflammatory and anticarcinogenic effects [44]. GLA specifically reduces inflammatory cytokines such as IL-1β, IL-6, and tumor necrosis factor (TNF)-α [44].

Of the 18 studies, 1 (6%) RCT evaluated the efficacy of GLA on AD severity and found significant improvement in symptoms from baseline in the intervention group, with no significant change in the control group [13]. However, the difference between these 2 groups was not significant, thus restricting the conclusions that can be drawn from this study [13]. Further limitations include the small sample size and the inclusion of patients with only mild to moderate AD, which may make it more difficult to ascertain meaningful symptom improvement in those with less severe symptoms. In addition, the lack of change in the use of topical corticosteroids between the groups also suggests the limited efficacy of GLA as a therapy [13].

Hempseed oil also has high concentrations of the essential fatty acids LA and α-LA in addition to the biologically active metabolites GLA and stearidonic acid [14]. These polyunsaturated fatty acids are present at an omega-6 to omega-3 ratio of 2.1:1, which may have various beneficial effects on human health, including reducing inflammation and reducing the risk of colorectal cancer [45]. It is hypothesized that an imbalance between omega-3 and omega-6 fatty acids contributes to the atopic and inflammatory responses observed in AD [46].

A single crossover study evaluated the effects of daily hempseed oil compared with olive oil on subjective AD symptoms, finding significant improvement in skin dryness and pruritus after 4 weeks of the intervention [14]. However, there was no significant difference between the olive oil and hempseed oil groups [14]. This study was limited by its small sample size, short study period, and lack of controls. The low power of the study and lack of significant intergroup findings make it difficult to accurately assess the efficacy of hempseed oil on AD, and larger, more rigorous studies are needed to assess polyunsaturated fatty acids’ true effect on AD.

### Probiotics

Probiotics are live microorganisms thought to restore the normal balance of the intestinal gut flora. Probiotics consist of many different bacterial species and strains, most commonly belonging to the *Lactococcus, Saccaromyces,* and *Bifidobacterium* genera [47]. Studies conducted on mice and skin models have shown that probiotics attenuate immune dysregulation through the inhibition of inflammatory cytokines and improved skin hydration [47]. Patients with AD also have abnormal intestinal microflora when compared with healthy patients, with lower concentrations of *Bifidobacterium* and higher concentrations of *Staphylococcus* [48]. Probiotics are thought to be beneficial for patients with AD because of their ability to restore the normal gut microbiome, but whether the atypical flora is the cause or a result of AD remains a controversial topic.

*Lacticaseibacillus paracasei* K71 decreases IgE synthesis both in vitro and in vivo [15]. A single RCT evaluated the effects of this strain on patients with AD for 12 weeks, finding improved skin severity, VAS, and quality of life impairment scores in the intervention group when compared with baseline but with no significant difference in improvement when compared with the placebo group. The limitations of this study include the small sample size and lack of controls.

Dysregulation of the immune system because of an imbalance of Th-1, Th-2, Th-17, and Foxp3 Treg cells is a key component of the pathological process of AD [16]. There is evidence supporting an increased production of IL-10 and a reduction in IgE, TNF-α, IL-5, and IL-17 in those taking probiotics [49,50]. *Lactiplantibacillus plantarum* IS-10506 improves Th-1 and Th-2 cytokine profiles by stimulating the intestinal microbiota through modulation of toll-like receptors, suggesting the possibility of alleviating AD symptoms [16].

Of the 18 studies, 2 (11%) separate RCTs evaluated the effectiveness of daily *L plantarum* on SCORAD scores for 8 weeks. Both showed that SCORAD scores significantly improved in the treatment group from baseline [16,17]; however, only 1 study found a significant difference in SCORAD scores in the intervention group when compared with the control group [16]. Important limitations include a multitude of confounding factors in both studies in addition to the small sample size and short observation period. However, the results provide promising evidence supporting the short-term efficacy of this intervention. The lack of a significant intergroup difference in both studies weakens the findings but could be the result of an underpowered study size and supports more extensive research into this specific strain.

Of the 18 studies, 2 (11%) separate studies evaluated the efficacy of another *Lactobacillus* strain, *L salivarius*. This strain decreases allergen-induced respiratory hyperresponsiveness and increases interferon-γ levels [51]. One trial evaluated the effectiveness of this strain alone, whereas the other used a dual combination of probiotic strains that included *S thermophilus* [18,19]. Both studies found significant improvement in SCORAD scores from baseline; the latter study also found significant differences in the intervention group compared with the control group [18,19]. The latter study also used a higher dose of *L salivarius* with the additional probiotic strain in combination with tara gum [19]. This combination has been shown to form a gel complex that adheres to the gastric mucosa and enhances intestinal barrier function [19]. A month after the intervention was stopped, the treatment group continued to show significant improvement in SCORAD scores when compared with the controls [19]. These promising results provide an impetus for further study of *L salivarius* and its use with *S thermophilus* and tara gum. However, the limitations of these studies include the lack of data on concurrent topical therapy or other treatments. In addition, the small sample sizes and lack of follow-up data 4 weeks after discontinuation make it difficult to gauge long-term efficacy.

An additional *Lactobacillus* strain, *L acidophilus*, was also tested for efficacy in patients with AD in 11% (2/18) of the studies: one evaluating the strain alone and one adding *B animalis lactis* [20,21]. Patients in the pure *L acidophilus* trial showed significant improvements in IGA, EASI, and SCORAD scores after 24 weeks compared with the placebo group [21]. Participants in the dual probiotic intervention trial were divided into groups based on total IgE levels (exogenous: total IgE level of >100 IU/mL; endogenous: total IgE level of <100 IU/mL) [20]. Patients were then further stratified according to the genotypes of the CD14 receptor gene, CC and TT [20]. The CD14 receptor gene locus on chromosome 5q31.1 contains the genes responsible for the synthesis of IgE [20].

This study found the most significant improvements in SCORAD and DLQI scores compared with the placebo group and the other intervention groups in participants with the exogenous form of AD and the CC genotype [20]. However, improvements in SCORAD and DLQI scores were noted from baseline in the endogenous and TT genotype groups as well, though these did not have significant intergroup differences [20].

A possible explanation for the differences between the endogenous and exogenous groups and patients with the TT and CC genotypes is an increased type-II immune response in patients with exogenous AD and the CC genotype [52]. Genetic polymorphisms and the immune system response to micro-organisms may contribute to the skin inflammation observed in AD, and part of this response is thought to stem from the activation of the CD14/ and TLR4 receptor complex by endotoxins of Gram-negative bacteria [20]. Various polymorphisms, including CC and TT (homozygous cytosine and thymine, respectively), affect the development of atopic disease, with studies showing that the number of positive skin tests, the risk of atopy, and the level of total IgE are increased in individuals with the CC genotype compared with those with the TT genotype [52-54]. Probiotics reduce total IgE, reduce inflammation, and stimulate regulatory T cells, thus inhibiting Th-2 cells and reducing TNF-α levels, mast cell degranulation, and eosinophil proliferation [55]. This is accomplished by probiotic lactic acid bacteria that enhance the Th-1 response and stimulate anti-inflammatory cytokines such as IL-10 and Transforming growth factor beta- [21].

*Bifidobacterium* is an additional genus of probiotics purported to have various beneficial effects on human health, including influencing the immune system by modulating the adaptive and innate immune responses, limiting pathogen colonization and invasion, and improving gut homeostasis [56]. One placebo-controlled trial looked directly at *B* *bifidum*’s effect on SCORAD scores, and there was no improvement with this intervention compared with the baseline or control group [17].

Another 11% (2/18) of trial studies examined the effectiveness of a combination of probiotic strains, including both *Lactobacillus* and *Bifidobacterium* [22,23]. Decreased levels of numerous intestinal bacteria, including *Bifidobacterium*, *Akkermansia,* and *Faecalibacterium*, may be related to early-onset AD [57]. Thus, these studies hoped that repopulating multiple strains, such as *Lactobacillus*, which can directly colonize microflora, and *Bifidobacterium*, which is a normally abundant genus in the human gut, would ameliorate AD symptoms [58]. These growth-promoting effects have the potential to exert anti-inflammatory action owing to the production of short-chain fatty acids such as propionate, acetate, and butyrate by numerous bacterial species [23]. The study evaluating the effects of combination probiotics with the addition of prebiotics found a significant change in EASI scores from baseline, and the trial evaluating combination probiotics alone found improvement in subjective symptoms to a greater degree in the intervention group, but this difference was not significant [22,23].

These trials do little to advance knowledge of these specific interventions because of their extremely small sample sizes and the inclusion of numerous probiotic strains and prebiotics, which confound the effective component of treatment. More research is needed into these combination therapies to determine their overall efficacy and effectiveness compared with single-strain probiotic supplements.

### Conclusions

Oral supplements continue to surge in popularity, with patients often turning to these over-the-counter options as medical alternatives for treating and alleviating AD symptoms. These supplements are not regulated by the Food and Drug Administration and, thus, do not have to meet the same safety or efficacy criteria that drugs do before entering the market. Therefore, to provide patients with accurate and up-to-date information, it is fundamental that medical professionals are aware of the current clinical data available regarding oral supplements.

Overall, there is weak evidence supporting any one nutritional supplement intervention for the alleviation of AD symptoms. Multiple trials (4/18, 22%) showed promise for supplements such as Zemaphyte, kefir, and freeze-dried whey with *C campestris* Yuncker extract; however, the small sample sizes and lack of controls in many of these trials make larger, higher-powered RCTs a necessity for determining the true value of these interventions. The most evidence was found on the efficacy of probiotics on the clinical course of AD; numerous studies (9/18, 50%) evaluated a multitude of bacterial probiotic strains, with many showing significant promise in improving AD symptoms. *Lactiplantibacillus plantarum*, *L salivarius*, and *L acidophilus* specifically showed evidence of efficacy and safety across multiple studies (6/18, 33%), but there is weak evidence supporting their use as an adjunctive treatment for AD. There is a need for larger, more extensive RCTs to determine the true effectiveness of these supplements on the broader population.

## Appendix

Multimedia Appendix 1 PRISMA Checklist.

## Abbreviations

25(OH)D: 25-hydroxyvitamin D
AD: atopic dermatitis
CFU: colony-forming unit
DLQI: Dermatology Life Quality Index
EASI: Eczema Area and Severity Index
GLA: gamma-linolenic acid
IGA: Investigator Global Assessment
IgE: immunoglobulin E
IL: interleukin
IU: international unit
LA: linolenic acid
PRISMA: Preferred Reporting Items for Systematic Reviews and Meta-Analyses
RCT: randomized controlled trial
SCORAD: Scoring Atopic Dermatitis
Th: T helper cell
TIS: Three-Item Severity
TNF: tumor necrosis factor
VAS: visual analog scale

## Data Availability

This protocol was registered and can be accessed at PROSPERO with the registration number CRD42023470596. The data used for the analysis can be obtained from IW by request.
